# Editorial: Angiogenesis and Angiogenesis Inhibitors in Oral Cancer

**DOI:** 10.3389/froh.2021.816963

**Published:** 2021-12-15

**Authors:** Abdelhakim Salem, Elin Hadler-Olsen, Ahmed Al-Samadi

**Affiliations:** ^1^Department of Oral and Maxillofacial Diseases, Clinicum, Faculty of Medicine, University of Helsinki, Helsinki, Finland; ^2^Translational Immunology Research Program (TRIMM), Research Program Unit (RPU), University of Helsinki, Helsinki, Finland; ^3^Department of Medical Biology, Faculty of Health Sciences, UiT the Arctic University of Norway, Tromsø, Norway; ^4^The Public Dental Health Service Competence Center of Northern Norway, Tromsø, Norway

**Keywords:** oral cancer (OC), angiogenesis, angiogenesis inhibitor, head and neck squamous cell carcinoma (HNSCC), vasculogenic mimicry (VM), salivary duct carcinoma (SDC), oral squamous cell carcinoma

This special issue of *Frontiers in Oral Health* sheds light on recent research regarding the tumor-related angiogenesis in oral cancer, including the present challenges of anticancer therapy targeting angiogenesis. Indeed, angiogenesis is a hallmark of solid tumors and it has important prognostic and therapeutic implications in cancer patients. This issue covers different relevant topics ranging from characterizing angiogenic signaling pathways to newly suggested paradigms of tumor neoangiogenesis revealing new putative therapeutic targets.

Oral squamous cell carcinoma (OSCC) is a common cancer worldwide, and the incidence has been rising steadily. Of great concern is the growing proportion of younger patients without a history of smoking or alcohol abuse among OSCC patients. Currently, the primary treatment of OSCC patients comprises surgery and (chemo) radiotherapy, either alone or in combination. Other clinically approved approaches, such as targeted- and immuno-therapies, remain inconsistently applied as a first-line treatment for OSCC patients. Unfortunately, despite the advancement in cancer management, these treatments offer limited efficacy and the 5-year survival rate among OSCC patients is around 50–60% [1, 2].

Angiogenesis is a key process in the tumorigenesis of solid cancers, promoting tumor growth, recurrence and metastasis. A large body of evidence suggested that targeting angiogenesis could provide a clinical benefit in the treatment of solid tumors [3]. Thus, the US Food and Drug Administration (FDA) approved the first angiogenesis inhibitor for cancer treatment—bevacizumab (Avastin®)—in February 2004. Bevacizumab, a recombinant monoclonal antibody targeting all isoforms of vascular endothelial growth factor (VEGF), has been approved initially for treating patients with metastatic colorectal cancer in combination with chemotherapy [4]. Since then, bevacizumab has initiated a new era in anticancer therapy, and anti-angiogenic drugs are now indicated for treating different malignancies including glioblastoma, breast, lung, ovarian, and cervical cancers. However, there are no FDA-approved anti-angiogenic drugs for head and neck squamous cell carcinoma (HNSCC) including OSCC, although these cancers arise in highly vascularized tissues [3, 5].

In spite of the promising results from some preclinical models, anti-angiogenic agents have revealed a considerable toxicity and limited clinical success in HNSCC patients [6]. This could be attributed, at least in part, to various tolerance mechanisms including an intrinsic or acquired drug resistance and the formation of tumor cell-derived mimetic vessels [3, 7]. In this Research Topic, Salem and Salo reviewed the vasculogenic (or vascular) mimicry (VM), a new paradigm of tumor cell-associated neovascularization that was introduced at the end of the previous millennium. VM describes the ability of certain aggressive tumors to initiate perfusable, matrix-rich, vessel-like networks in collagen-rich matrices *in vitro* and *in vivo*. This phenomenon has ignited a vibrant research interest and revealed promising prognostic and therapeutic implications. Further, it provides a possible explanation of the modest anti-angiogenic drug response in cancer patients. This review summarized the available knowledge regarding VM formation in HNSCC ranging from the characterization protocols to the prognostic value and limitations. Finally, the authors presented a potential novel mechanism of lymphatic metastasis in HNSCC—lymphatic mimicry (LM). Mirroring VM, this concept describes a process whereby tumor cells form mosaic vessel-like structures expressing tumor and lymphatic cell markers in OSCC tissues and *in vitro*. These LM structures influenced tumor cell invasion and metastasis and could provide avenues for developing anti-lymphangiogenic drugs in HNSCC [8].

In their research on medicinal natural compounds, Pang et al. showed that GT198, a protein inducing angiogenesis in OSCC, can be targeted by anticancer agents and herbs. Of interest, several clinically valid drugs inhibited the activity of GT198, including but not limited to mitoxantrone, doxorubicin and imatinib. A strong inhibition was further seen when applying certain anticancer herbs such as allspice, *Gleditsia sinensis* or honey locust. These findings could open an opportunity to identify potential antiangiogenic natural drugs with high efficacy and low toxicity for treating patients with oral cancer.

Although OSCC is the most common tumor type in the head and neck region, other less frequent, yet highly aggressive, tumors can also be encountered in the oral cavity. In this regard, Suzuki et al. studied an angiogenesis-associated pathway in salivary duct carcinoma (SDC), a cancer that can arise as a primary tumor (*de novo*) or from recurrent carcinoma ex-pleomorphic adenoma (Ca-ex-PA). Similar to the ductal breast carcinoma, SDC is characterized by an induced human epidermal growth factor receptor 2 (HER2) status. In the study included in this Research Topic, authors conducted immunostaining and dual color *in situ* hybridization of HER2 (Di Palma Classification) for patients with SDC, and analyzed the expression levels of 84 functional genes using PCR. Of note, significant differences in gene expression were found between Ca-ex-PA and *de novo* tumors. VEGF-A was significantly induced in the Ca-ex-PA, which can be a crucial factor in the malignant conversion to SDC through angiogenesis and the AKT/PI3K signaling pathway. Multiple machine learning methods confirmed that the prominent proangiogenic factor VEGF-A can be a candidate for differentiating Ca-ex-PA from *de novo* lesions.

Since oral cancer continues to constitute a substantial health and economic burden worldwide, we hope that this Research Topic will be informative to clinicians, researchers, and health care providers.

## Author Contributions

All authors listed have made a substantial, direct, and intellectual contribution to the work and approved it for publication.

## Conflict of Interest

The authors declare that the research was conducted in the absence of any commercial or financial relationships that could be construed as a potential conflict of interest.

## Publisher's Note

All claims expressed in this article are solely those of the authors and do not necessarily represent those of their affiliated organizations, or those of the publisher, the editors and the reviewers. Any product that may be evaluated in this article, or claim that may be made by its manufacturer, is not guaranteed or endorsed by the publisher.
